# The Heart Health Study - increasing cardiovascular risk assessment in family practice for first degree relatives of patients with premature ischaemic heart disease: a randomised controlled trial

**DOI:** 10.1186/s12875-015-0328-4

**Published:** 2015-09-03

**Authors:** Nigel P. Stocks, Jessica L. Broadbent, Michelle F. Lorimer, Philip Tideman, Derek P. Chew, Gary Wittert, Philip Ryan

**Affiliations:** Discipline of General Practice, NHMRC Centre of Research Excellence to Reduce Inequality in Heart Disease, University of Adelaide, Adelaide, South Australia Australia; Data Management and Analysis Centre, University of Adelaide, Adelaide, South Australia Australia; Department of Cardiovascular Medicine, Flinders University, Bedford Park, South Australia Australia; School of Medicine, University of Adelaide, Adelaide, South Australia Australia; School of Population Health, University of Adelaide, Adelaide, South Australia Australia

## Abstract

**Background:**

This study aimed to increase cardiovascular disease (CVD) risk assessment in adult first degree relatives of patients with premature ischaemic heart disease (PIHD) using written and verbal advice.

**Methods:**

*Design*: A prospective, randomised controlled trial. *Setting*: Cardiovascular wards at three South Australian hospitals. Cardiovascular risk assessments were performed in general practice. *Participants*: Patients experiencing PIHD (heart disease in men aged <55 years or women aged < 65 years) and their first degree relatives. *Intervention*: Patients distributed either general information about heart disease and written advice to attend their general practitioner (GP) for CVD risk assessment or general information about heart disease only, to their first degrees relatives. *Main outcome measure*: The primary outcome was the proportion of relatives who attended their GP for CVD risk assessment within 6 months of the patients’ PIHD event.

**Results:**

One hundred forty four patients were recruited who had 541 eligible relatives; 97/541 (18 %) of relatives agreed to participate. A larger number of intervention 41/55 (75 %) than control group 9/42 (21 %) [difference 53 %, 95 % CI 36 % - 71 %] relatives attended their GP for a CVD assessment, and 34 % of these had moderate to very high 5-year absolute risk for CVD.

**Conclusion:**

This low cost intervention demonstrates that individuals who have a family history of PIHD and are at moderate or high risk of CVD can be targeted for early intervention of modifiable risk factors. Further research is required to improve the uptake of the intervention in relatives.

**Trial registration:**

The trial was registered with the Australian Clinical Trials Registry (ACTRN), Registration ID 12613000557730.

## Background

Cardiovascular disease (CVD) accounted for 34 % of all deaths and 12 % of the total allocated health system expenditure ($7.6 billion) in Australia in 2008 [1]. The most common form of CVD, ischaemic heart disease (IHD), was estimated to effect 3 % (685,000) of Australians in 2007–2008 [2]. First degree relatives of patients with premature IHD (PIHD), defined as males aged < 55 years, or females < 65 years, are at increased risk of CVD [3–5] and are an ideal target for primary preventive measures [6].

The Joint Task Force of European and other Societies on Coronary Prevention advised in 1994 and 1998 that close relatives of patients with PIHD should be screened for coronary risk factors [7, 8] and the US National Cholesterol Education Program III (NCEP) also recommends screening patients with a recognised family history [9]. However evidence from Europe and the US indicates that relatives of patients with PIHD are being overlooked in primary prevention and several studies have highlighted the potential benefits of focusing on high-risk families [10, 11]. In the EUROASPIRE II family survey [12], self-reported family screening occurred in only 11.1 % of siblings and 5.6 % of children of 1289 index patients with PIHD.

The idea for this study arose from conversations amongst a sub-group of investigators about successful interventions aimed at known risk factors for IHD such as smoking, hypertension and hyperlipidemia, and the relative lack of studies concerning PIHD. At the commencement of this study, there were no published Australian guidelines that promoted screening for families experiencing PIHD and at least anecdotally, no system for alerting general practitioners (GPs) about familial risk. Targeting primary prevention at families with a positive family history was seen to be a potentially cost-effective strategy to tackle the burden of IHD in Australia and all developed countries. This study aimed to ascertain if the provision of written and verbal advice, promoting cardiovascular risk assessment to first-degree relatives of patients with PIHD, increased the proportion of relatives undertaking cardiovascular risk assessment in general practice.

## Methods

### Trial design

A prospective, multicentre, randomised controlled trial conducted in Adelaide, South Australia.

### Ethics approval

The study was ethically approved by the Royal Adelaide Hospital Research Ethics Committee and Flinders Clinical Research Ethics Committee, and approved for conduct at Flinders Private Hospital. All participants provided their written, informed consent for participation in this study.

### Inclusion and exclusion criteria

Men aged < 55 and women aged < 65 hospitalised with their first PIHD event. An IHD event was defined as any one of the following: non-ST-segment elevation myocardial infarction (NSTEMI), ST-segment elevation myocardial infarction (STEMI), unstable angina or coronary revascularisation (coronary artery bypass grafting, coronary angioplasty with or without coronary stenting).

First degree relatives (siblings and children) of the patient aged at least 18, without diagnosed IHD and residing in Australia.

Patients and relatives were excluded if they were: terminally ill, experiencing dementia and/or other significant cognitive impairment, unable to speak English, illiterate, had any condition that their treating doctor believed warranted intensive family follow-up and intervention, or Aboriginal and living in a remote indigenous community.

### Participant recruitment

Patients were recruited from tertiary care cardiovascular wards at the two largest tertiary teaching hospitals in South Australia: the Royal Adelaide Hospital, Flinders Medical Centre and Flinders Private Hospital from July 2009 to February 2012, and were approached only after discharge from coronary care units into a ward or were stable after a revascularisation procedure.

Research nurses at each site approached patients hospitalised with PIHD, discussed the study, provided written information and obtained written, informed consent. Potential participants were advised that the study was about cardiovascular risk assessment in their first degree relatives, and that by agreeing to participate in the study that they would be required to forward study information to their relatives. The nurse constructed a family tree with information provided by the patients. Data collected included: relative status (brother, sister, son, daughter), age, initials, parent’s initials and age, and if the relative had a diagnosis of IHD.

### Treatment allocation

Following receipt of written consent from each patient, the research nurse selected the next sequentially numbered, opaque and sealed envelope containing a computer generated treatment allocation. Randomisation was conducted for each recruitment site by a statistician not involved in the study analyses. Random number generation, with a 1:1 ratio using SAS (version 9.1 SAS Institute, Cary, NC, USA) [13].) was based on a sample size of 140 (accommodating up to 80 possible patient recruits at each site). Patients were randomly allocated to provide either the ‘intervention’ or the ‘control’ information packs to all of their participating first degree relatives. Packs were given by the research nurse to the patients, who then distributed them to relatives whilst still in hospital or posted them to relatives at a later date. All patients were blinded as to which information pack they were providing to their relatives.

#### Intervention group

Intervention group relatives were provided with a consent form, reply paid envelope and information sheet that explained the reason for the study and provided a brief explanation about PIHD, cardiovascular risk factors and the benefits of risk factor assessment. It included a recommendation that the relative make an appointment to attend their general practitioner (GP) for a cardiovascular risk assessment (without cost to them). Completion and return by the GP to the study center of a post-card included in the study pack was taken as evidence of attendance. Using the participant’s age, sex, blood pressure (mmHg), diabetes status, smoking status and total cholesterol: HDL ratio as recorded by the GP, a 5-year absolute CVD risk value was calculated and multiplied by 1.5 (e.g. 10 % increased to 15 %) to account for the pre-existing family history of premature heart disease in all study participants [5]. The CVD risk factor chart, placed on the reverse side of the postcard, was derived from the New Zealand Guidelines Group, Heart Foundation, Stroke Foundation and NZ Ministry of Health [14] and is based on Framingham Study data. The 5 year CVD risk was calculated by the GP and used in their consultation with the GP, however the risk was recalculated by research staff when the postcard was returned, and this % used for data analysis.

Additionally, intervention relatives were advised to contact the Heart Foundation for free telephone advice about heart disease, and were given the option to receive further telephone advice from a study doctor or study nurse, according to their preference.

#### Control (usual care) group

Control group relatives were provided with a consent form, reply paid envelope and information sheet that explained the reason for the study and provided a brief explanation about PIHD and cardiovascular risk factors only. It did not include a recommendation to attend their GP for a cardiovascular risk assessment, contact the Heart Foundation or the option of further advice about heart disease from a doctor or nurse.

### 6 month follow-Up

Six months after consent, all consenting relatives were contacted to assess events following their relative’s hospitalisation with an IHD event (during the January 2010 to August 2012 follow-up period). The brief telephone conversation ascertained whether in the last 6 months there had been: GP attendance with a cardiovascular risk assessment performed, any cardiovascular risk factors identified, any lifestyle changes made and how these had been managed by their GP. A brief follow-up questionnaire asking the same series of questions was posted to participants unable to be reached by telephone, following two attempts. After follow-up, control group relatives were subsequently provided with the same written information given to the intervention group.

### Outcomes

The primary outcome of the study was the proportion of relatives who attended their GP for cardiovascular risk assessment within 6 months of the patients’ PIHD event. Post card return and self-reported attendance in the intervention group were compared with self-reported attendance in the control group. A secondary outcome of the study was the absolute cardiovascular risk of relatives in the intervention group, calculated from the GP supplied information on the returned postcard.

### Sample size and statistical analysis

#### Sample size

The sample size calculation made the following assumptions:

- 30 % of patients would agree to participate in the study (of these approximately 10 % will die or otherwise not be able to participate in the first 6 months of the study)
- each patient had on average, three first-degree relatives (inclusive of sibling(s) and children)
- 20 % of relatives would be ineligible due to an existing IHD diagnosis and 50 % of eligible relatives would agree to participate in the study, allowing for a design effect of 1.1 (using a family intra-cluster correlation coefficient of 0.05, and an average cluster size of 3, the design effect [15] is 1 + (3–1)*0.05 = 1.1)
- A maximum dropout rate for relatives in the order of 10 % over the course of the study.

A total minimum sample size of 140 patients and 136 relatives was calculated as sufficient to detect an increase of 25 % (absolute) in general practitioner attendance for IHD risk-assessment, with 25 % of control group relatives attending in the 6 months after the event, compared with 50 % of relatives in the intervention group, and allowing for a correlation of 0.25 with other covariates (e.g. age, gender), with at least 80 % power (90 % power for a one sided test) at the 5 % significance level.

#### Statistical analysis

We present means and frequencies to describe the baseline characteristics of both patients and relatives assigned to the control and intervention study groups. We used a generalised linear model with an identity link and binomial error, and generalised estimating equations, to estimate the difference in proportions of relatives attending their GP between two randomised groups, allowing for clustering within family. No adjustments we made for baseline covariates. Statistical analyses were performed using SAS version 9.1 (SAS Institute, Cary, NC, USA) [13].

The authors of this paper had full access to all of the data (including statistical reports and tables) in the study.

## Results

### Participants

#### Patients

**Fig. 1:**
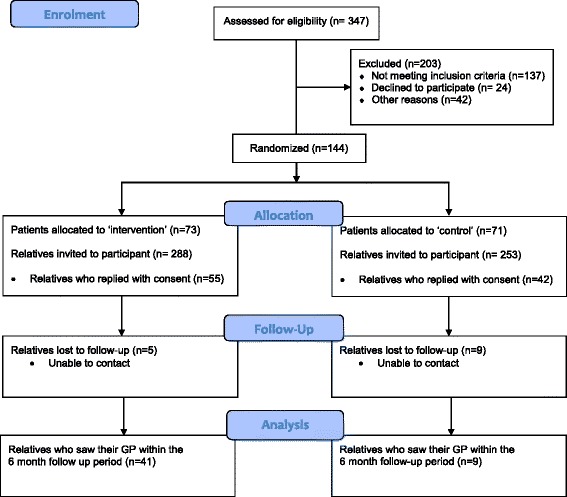
Consort flow diagram for the heart health study

From 347 patients approached for inclusion 144 (42 %), aged between 26 and 63 years, 33 % female and 67 % male, provided their written informed consent for participation and were randomly allocated to intervention (*n* = 73) or control (*n* = 71) arms. The remaining 203 (58 %) patients were not enrolled because either they did not meet the inclusion criteria *n* = 137 (67 %), of whom *n* = 61 had pre-existing heart disease, declined to participate *n* = 24 (12 %) or did not participate for other reasons *n* = 42 (21 %) which included 28 people living in remote Aboriginal communities (Fig. 1).

#### Relatives

Patients collectively identified 541 first degree relatives who met the inclusion criteria and could be invited for participation in the study. A total of 288 intervention and 253 control information packs were provided to these relatives. Feedback at recruitment and at the 6 month follow-up indicated that some patients would not send packs to all their relatives because they were estranged from some or all of their siblings or children.

Ninety seven (97) relatives responded with their consent for participation, intervention (*n* = 55) and control (*n* = 42). Overall 18 % of relatives invited to participate replied with consent (19 % and 17 % of the intervention and control group relatives, respectively). Six month follow-up telephone calls (or postal questionnaire, if unable to reach after 2 attempts) were completed for 93 % (51/55) and 79 % (33/42) for the intervention and control groups, respectively. Refer Table 1 for participant summary.

**Table 1:** Summary of participating patients with PIHD (*n* = 144) and their first degree relatives (*n* = 97)

	Intervention	Control	Total
Patients, n	73	71	144
Male	50	46	96 (67 %)
Female	23	25	48 (33 %)
Age, range	26-63	30-65	26-65
Age, mean (SD)	49 (6.13)	51.1 (6.5)	50 (6.4)
First degree relatives, n (%)			
*Invited*	288	253	541
- Sister	91 (32 %)	79 (31 %)	
- Brother	93 (32 %)	72 (28 %)	
- Daughter	54 (19 %)	60 (24 %)	
- Son	50 (17 %)	42 (17 %)	
*Replied with consent*	55 (19 %)	42 (17 %)	97 (18 %)
- Sister	26	16	
- Brother	20	7	
- Daughter	5	12	
- Son	4	7	

### GP attendance for cardiovascular risk assessment

**Fig. 2:**
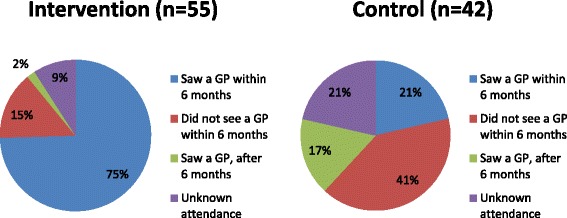
Proportion of relatives who attended their GP for a cardiovascular risk assessment, Intervention (*n* = 55) versus Control (*n* = 42)

A larger number of intervention (75 %; 41/55) than control group (21 %; 9/42) [difference 53 %, 95 % CI 36 % – 71 %] relatives attended their GP for a cardiovascular risk assessment within 6 months of consent (Fig. 2). A greater proportion of control (41 %; 17/42) than intervention group (15 %; 8/55) relatives did not see their GP at all during the 6 months study follow-up. A small number of control (*n* = 7) and intervention (*n* = 1) group relatives visited their GP after being contacted by the research team at 6 months (ascertained from late return of the GP postcards after the 6 month follow-up). Overall 14 % (41/ 288) of relatives in the intervention group attended for cardiovascular risk assessment compared with 3.5 % (9/253) of control group relatives.

### Absolute cardiovascular risk

**Fig. 3:**
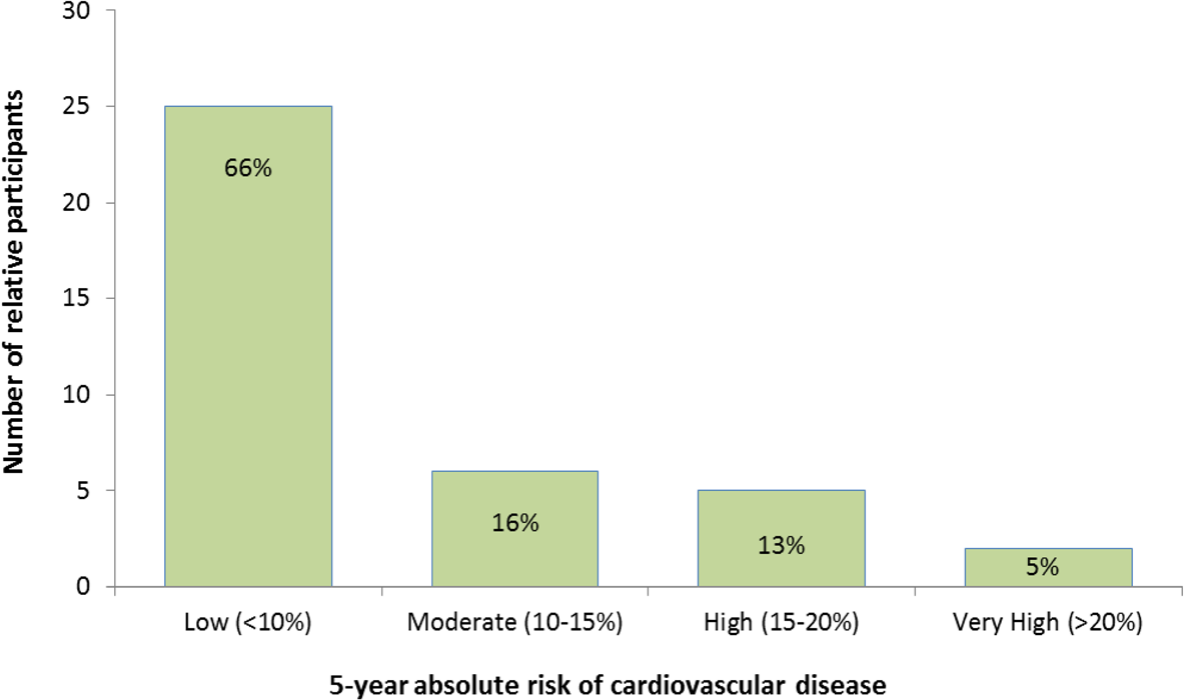
5 year absolute risk of cardiovascular disease (Intervention *n* = 38)

A 5-year absolute CVD risk was calculated for all intervention relatives who visited their GP during the 6 month follow-up period for whom a completed post-card was returned (*n* = 38). The majority of relatives who attended their GP for a cardiovascular risk assessment had low absolute cardiovascular risk (66 %) (Fig. 3); five-year absolute CVD risk was moderate, high or very high in the remaining 34 %. All moderate to very high risk relatives were identified to be siblings (not children) of the PIHD patients.

## Discussion

The results of this study demonstrate that when simple written advice is given to first degree relatives of patients with PIHD 75 % undertake GP cardiovascular risk assessment within 6 months compared to 21 % in the control group. In addition 34 % of relatives, all siblings, were found to be at moderate to very high risk of CVD in the next 5 years, which has important implications for primary prevention in this group.

Although published guidelines recommend screening of first degree relatives of patients with PIHD [16, 17] none appear to have evidence based strategies for achieving this in practice. Screening tends to be opportunistic [12] and there is evidence that siblings and children are missing out [10]. Based on our findings we would recommend that such advice for relatives should be part of cardiac rehabilitation and when this is not available, be part of a discharge package given to all patients with PIHD. Although it might be tempting to focus only on siblings of patients, who tended to have a higher absolute CVD risk, we believe that for children this might be an important moment for behavioral change which should be reinforced by visit to a general practitioner.

The feasibility of implementing family screening in a hospital setting has been examined in a European study [3]. Their results were similar to ours with 63.9 % of relatives in the intervention group undergoing CVD risk assessment compared to 25.4 % in the control group. Whilst their participation rate was higher this may reflect the clustered study design, the role of enthusiastic cardiologists as part of their intervention and potentially how people access care in the Belgian health system.

Our intervention was successful in getting some moderate and high CVD risk individuals to have a cardiovascular risk assessment. Guidelines and review papers recommend the use of absolute cardiovascular risk to guide management of risk factors [18] and a reduction in population absolute cardiovascular risk has been demonstrated in an RCT of health checks [19]. Providing information to a first degree family member shortly after a relative’s recent heart event could make the most of a window of opportunity, where receptivity to a new health message may promote ‘contemplation’, leading to readiness and ‘preparation’ to take ‘action’, as identified in the Transtheoretical Model of Health Behaviour Change [20]. There is, of course, the potential to do harm by generating anxiety in relatives who are actually at low risk. Given our finding that it was siblings who were found to be moderate or high risk it may be appropriate to limit dissemination of information to this group only.

It has been estimated that in the UK 1,011 myocardial infarctions (MI) could be prevented over 5 years if the siblings of patients, who had been admitted to hospital with an episode PIHD in one year, could be identified and managed with a ‘polypill’ [6]. Using Australian figures for MI and the assumption that PIHD accounts for 20 % of all CVD then the equivalent Australian totals (55,000 MIs/year) would be 712 preventable heart attacks over 5 years. Clearly the first step in preventing these heart attacks is identifying those at risk. Whilst these figures may be optimistic, our intervention appears to be a good investment in ‘best’ or at least ‘better’ practice.

Privacy and confidentiality laws usually limit the ability of hospitals and treating physicians to make direct contact with persons related to their patients. Our indirect method circumvents this issue; however we did find that some patients were estranged from either their siblings or children. This appears to be a common issue and will be difficult to overcome in clinical practice because it is hard to argue for overriding concerns about the future health of relatives when only a proportion are at moderate or high risk.

### Limitations

Because this study was conducted in two major teaching hospitals in Adelaide and the majority of relatives lived in South Australia the generalizability of the findings may be limited. The study did include a mix of public and private patients; however we cannot be sure that the socio economic or educational status of our participants truly reflects the general population. Ideally we would have collected such data on all patients and relatives however this had to be balanced against increasing the burden of paperwork in both groups and therefore lowering the participation rate.

Ideally we would have used reduction in mortality or at least absolute cardiovascular risk as the primary outcome measure but the additional follow-up and time required were beyond the resources of this study. We were interested in a ‘proof of concept’ that could be developed further if proven to be feasible.

Our study potentially incentivised the GP visit by offering to pay for any gap to ensure equity of access for all, however if the intervention was applied in practice any gap payment might reduce the number of relatives seeking GP CVD risk assessment. Considering that approximately 80 % of all GP service are currently bulk billed (at no cost to the patient) [21] the impact might be limited and in addition there is mixed evidence that financial incentives improve attendance rates for CVD risk assessment [22].

We assessed 347 hospitalised patients for eligibility and randomized 144. Clearly many ineligible patients (137), those living in remote aboriginal communities (28) and their first degree relatives would have a different set of circumstances from those participating in a trial. In addition there were some hospitalised patients who were not approached because they were recruited for other trials or were on the wards at weekends or holidays. As with any other trial we have selected an eligible population for whom the results are applicable and we cannot make any inferences about the impact of the intervention on these other groups who make up a substantial proportion of all PIHD patients and their relatives.

The participation rate of relatives was low but amongst all relatives who were eligible to participate at least 10 % (14 % versus 3.5 %) more in the intervention group attended for cardiovascular risk assessment. A result, if attendance for risk assessment is seen as beneficial and given the low cost of the intervention, that makes providing information to patients to distribute to their relatives worthwhile. The participation rates may have been low because: it was a research study that required consent, patients may not have forwarded the information on to relatives, or reflects a lack of interest in preventive health care. Equally relatives may have received the information and acted upon it without formally being part of the study. Unfortunately, due to ethical constraints, we could not send reminders or follow up non-responders.

## Conclusions

Providing simple written and verbal advice to patients hospitalised with PIHD to distribute to their adult children, brothers and sisters leads more people to have a cardiovascular risk assessment by their GP. Given the simplicity of the intervention, and the number of relatives at moderate or high CVD risk who could benefit from primary prevention, it is likely to be a cost effective way of reducing the burden of CVD in Australia and all developed countries around the world. More research is required to evaluate if the content of the information provided improves response rates, what mechanisms improve the distribution of information to first degree relatives and how to encourage more relatives to attend for CVD risk assessment.

### Availability of supporting data

Data will not be available at this stage.

## Abbreviations

CVD: Cardiovascular disease
GP: General practitioner (Family Physician)
IHD: Ischemic heart disease
NSTEMI: Non-ST-segment elevation myocardial infarction
PIHD: Premature ischemic heart disease
RCT: Randomised controlled trial
STEMI: ST-segment elevation myocardial infarction

## References

[1] Australian Institute of Health and Welfare (2014). Health care expenditure on cardiovascular diseases 2008–2009. Cat. no. CVD 65.

[2] Australian Institute of Health and Welfare (2011). Cardiovascular disease: Australian facts 2011. Cardiovascular disease series no. 35. Cat. no. CVD 53.

[3] Tonstad S, Westheim A (2002). Implementation of guidelines to screen relatives of patients with premature coronary heart disease in a hospital setting. Am J Cardiol.

[4] Qureshi AI, Suri MF, Guterman LR, Hopkins LN. Ineffective secondary prevention in survivors of cardiovascular events in the US population: report from the Third National Health and Nutrition Examination Survey. Arch Intern Med. 2001;161:1621–8. 10.1001/archinte.161.13.1621 11434794

[5] Myers RH, Kiely DK, Cupples LA, Kannel WB. Parental history is an independent risk factor for coronary artery disease: the Framingham Study. Am Heart J. 1990;120:963–9. 10.1016/0002-8703(90)90216-k 2220549

[6] Chow CK, Pell AC, Walker A, O'Dowd C, Dominiczak AF, Pell JP. Families of patients with premature coronary heart disease: an obvious but neglected target for primary prevention. BMJ. 2007;335:481–5. 10.1136/bmj.39253.577859.BE PMC1971158 17823190

[7] Pyörälä K, De Backer G, Graham I, Poole-Wilson P, Wood D. Prevention of coronary heart disease in clinical practice Recommendations of the Task Force of the European Society of Cardiology, European Atherosclerosis Society and European Society of Hypertension. Eur Heart J. 1994;15:1300–31. 10.1093/oxfordjournals.eurheartj.a060388 7821306

[8] Wood D, De Backer G, Faergeman O, Graham I, Mancia G, Pyörälä K. Prevention of coronary heart disease in clinical practice. Recommendations of the second joint task force of European and other societies on coronary prevention. Eur Heart J. 1998;19:1434–503. 10.1016/s0021-9150(98)90209-x 9862269

[9] Panel TE (1993). Summary of the second report of the National Cholesterol Educational Program Expert Panel on Detection, Evaluation, and Treatment of High Blood Cholesterol in Adults. JAMA.

[10] Hengstenberg C, Holmer SR, Mayer B, Engel S, Schneider A, Lowel H, *et al*. Siblings of myocardial infarction patients are overlooked in primary prevention of cardiovascular disease. Eur Heart J. 2001;22:926–33. 10.1053/euhj.2000.2413 11428816

[11] Swanson JR, Pearson TA (2001). Screening family members at high risk for coronary disease: Why isn’t it done?. Am J Prev Med.

[12] De Sutter J, De Bacquer D, Kotseva K, Sans S, Pyörälä K, Wood D, *et al*. Screening of family members of patients with premature coronary heart disease. Results from the EUROASPIRE II family survey. Eur Heart J. 2003;24:249–57. 10.1016/s0195-668x(02)00386-x 12590902

[13] SAS Institute. SAS/STAT User's Guide: Version 9.1. Cary, NC: SAS Institute, 2004.

[14] New Zealand Guidelines Group. New Zealand Cardiovascular Guidelines Handbook: A summary resource for primary care practitioners. Second edition. Wellington, New Zealand Guidelines Group; 2009. www.health.govt.nz/publication/new-zealand-cardiovascular-risk-charts (accessed Apr 2014).

[15] Kish L (1965). Survey Sampling.

[16] Wood D, Wray R, Poulter N, Williams B, Kirby M, Patel V, *et al*. JBS2: Joint British Societies’ guidelines on prevention of cardiovascular disease in clinical practice. Prepared by: British Cardiac Society, British Hypertension Society, Diabetes UK, HEART UK, Primary Care Cardiovascular Society, The Stroke Association. Heart. 2005;S5(91):v1–7. 10.1136/hrt.2005.079988 PMC1876394 16365341

[17] National Heart Foundation of Australia and the Cardiac Society of Australia and New Zealand (2012). Reducing risk in heart disease: an expert guide to clinical practice for secondary prevention of coronary heart disease.

[18] Nelson MR, Doust JA (2013). Primary prevention of cardiovascular disease: new guidelines, technologies and therapies. Med J Aust.

[19] Cochrane T, Davey R, Iqbal Z, Gidlow C, Kumar J, Chambers R, *et al*. NHS health checks through general practice: randomised trial of population cardiovascular risk reduction. BMC Public Health. 2012;12:944. 10.1186/1471-2458-12-944 PMC3524756 23116213

[20] Prochaska JO, Velicer WF (1997). The transtheoretical model of health behavior change. Am J Health Promot.

[21] Australian Government Department of Health. Annual Medicare Statistics – Financial Year 2007–08 to 2012–13.2013. www.health.gov.au/internet/main/publishing.nsf/Content/Annual-Medicare-Statistics.

[22] Stocks N, Allan J, Frank O, Williams S, Ryan P (2012). Improving attendance for cardiovascular risk assessment in Australian general practice: an RCT of a monetary incentive for patients. BMC Fam Pract.

